# Supplementary figures and images for: Short communication: Evaluation of charged membrane filters and buffers for concentration and recovery of infectious salmon anaemia virus in seawater
Source: PLoS One. 2021 Jun 16;16(6):e0253297. doi: 10.1371/journal.pone.0253297 (PMC8208535; doi:10.1371/journal.pone.0253297)

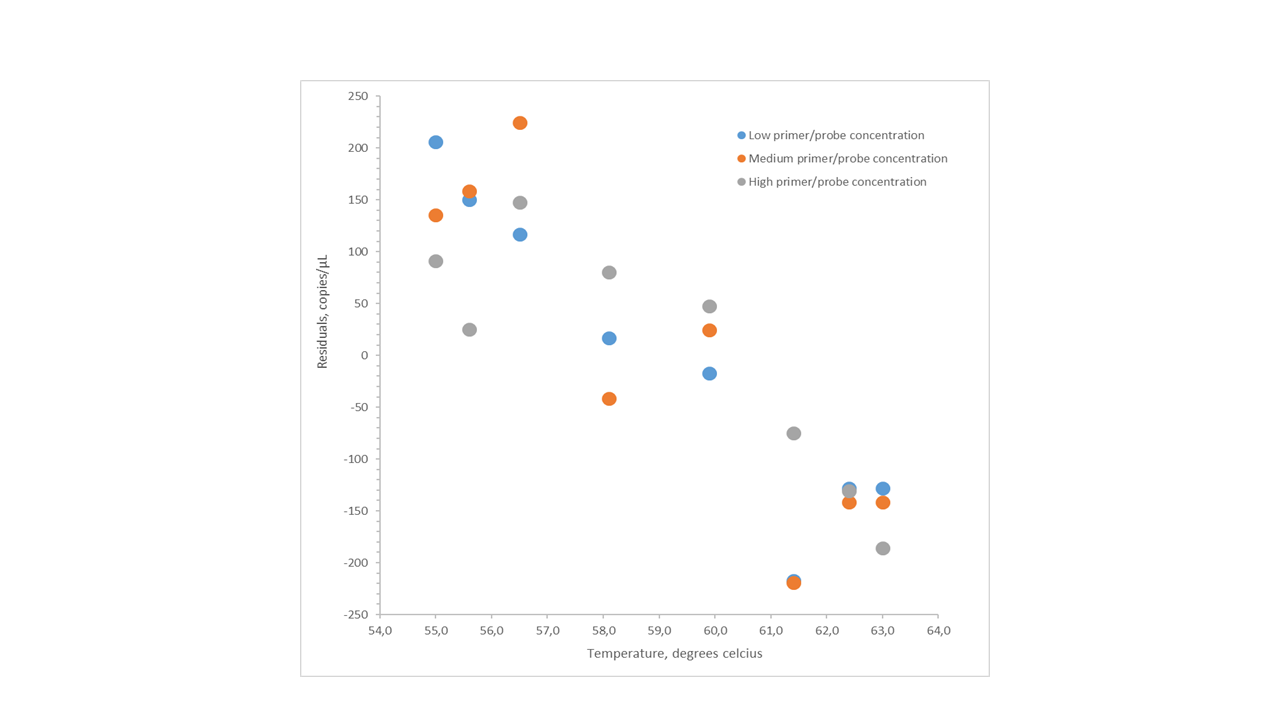

Supplement: S1 Fig — (TIF) [file pone.0253297.s001.tif]
