# Supplementary material for: Short communication: Evaluation of charged membrane filters and buffers for concentration and recovery of infectious salmon anaemia virus in seawater
Source: PLoS One. 2021 Jun 16;16(6):e0253297. doi: 10.1371/journal.pone.0253297 (PMC8208535; doi:10.1371/journal.pone.0253297)
Supplement: S1 Table — (DOCX) [file pone.0253297.s002.docx]

**S1 Table.** Show ISAV recovery (Residual) with the different annealing temperatures and primer/probe concentrations.

| **Temperature** | **Primer/probe concentration** | | |
| --- | --- | --- | --- |
|  | **Low (600/150)** | **Medium (Recommended; 900/250)** | **High (1200/350)** |
| 63.0 | -128 | -141.4 | -185.8 |
| 62.4 | -128 | -141.4 | -130.8 |
| 61.4 | -217 | -219.4 | -74.8 |
| 59.9 | -17 | 24.6 | 47.3 |
| 58.1 | 17 | -41.4 | 80.3 |
| 56.5 | 117 | 224.6 | 147.3 |
| 55.6 | 150 | 158.6 | 25.3 |
| 55.0 | 206 | 135.6 | 91.3 |
