# Supplementary material for: Short communication: Evaluation of charged membrane filters and buffers for concentration and recovery of infectious salmon anaemia virus in seawater
Source: PLoS One. 2021 Jun 16;16(6):e0253297. doi: 10.1371/journal.pone.0253297 (PMC8208535; doi:10.1371/journal.pone.0253297)
Supplement: Attachment — Submitted filename: Response to Reviewers.docx [file pone.0253297.s004.docx]

**PONE-D-21-01328**

Dear Editor,

Thank you for the positive feedback, and all the constructive comments from you and the reviewers to the manuscript: *Evaluation of Charged Membrane Filters and Buffers for Concentration and Recovery of Infectious Salmon Anemia Virus in Seawater*

**Authors:** Simon Chioma Weli, Haitham Tartor, Bjørn Spilsberg, Ole Bendik Dale, Atle Lillehaug

We have revised the manuscript as recommend by you and the two reviewers by addressing all the comments raised. We have attached the following files:

1. A revision showing edits made in this revision as tracked changes (in Word doc format), file name: ***“Revised Manuscript with Track Changes***”.
2. A revision with all changes accepted ('clean' file, in Word doc format), which is an unmarked version of the revised manuscript without tracked changes. File labeled ***“manuscript”.***
3. A response letter, file name: ***“Response to Reviewers”***, which is a rebuttal letter that responds to each point raised by the academic editor and reviewers.

**Editor’s comments:** As a comment to the standard text I would like to add that the paper needs minor revision in terms of the scientific content as suggested by reviewer 2. However, as reviewer 1 has commented, a similar paper has already been published with another virus (SAV) by your group. Please consider to shorten the current manuscript accordingly, referring on the SAV paper, stating the modifications of the methods described for SAV recovery from sea water. Actually, I ask myself why both manuscripts weren't combined in one paper.

***Author’s response:*** *As suggested by reviewer 1, we have shortened the manuscript and referred to the method as described for SAV recovery from seawater. With regard to why both manuscript (SAV and ISAV) were not combined in one paper. The reason was that, although the entire funding for the research was for developing a method for concentration and recovery of fish viruses (ISAV and SAV) from seawater, the SAV aspects of the research work was specifically organized as part of a PhD thesis. Combining both manuscript (SAV and ISAV) in one paper would be* *problematic for the student. It will mean that the PhD student would have to defend a subject that is not part of her PhD topic, as published articles derived from PhD thesis work are normally attached and defended during dissertation.*

**Journal Requirements:**

*We have revised the manuscript to meet PLOS ONE format and style as requested by the academic editor.*

*We have reviewed the reference. Change to the reference list: Reference #21 was updated to reflect correct article publication date*

**Reviewer #1 comment:** In my opinion, this work could not be accepted for publication in current form and should be revised highlighting the findings related to ISAV as short communication with reference to published work for methodology.

***Author’s response:*** *As suggested by reviewer 1, we have shortened the manuscript (as short communication) without omitting details that will allow readers to have full understanding of the work. We have also referenced the method as described for SAV recovery from seawater. The reduction where mainly focused on the materials and method section.*

**Reviewer #2:**

**Reviewer comment:** Line 134-137 Did the researchers include a positive control either directly in the RNA extraction or spiked at the lysis stage? This would help control for loss of RNA during extraction process.

***Author’s response:*** *No, we did not include* *positive control directly during RNA extraction or spiked at the lysis stage in this particular (in vitro) experiment. However, in our field study, we did add Mengovirus to evaluate extraction efficiency of SAV recovery from seawater. We agree that addition of spike positive control,* would have probably helped to check for loss of RNA during extraction process.

**Reviewer comment:** Line 141 – Only 1 ml of lysate was used for the RNA extraction. However, the volume of lysis buffer added to the filters was 4ml therefore only ¼ of the sample was extracted. Was the yield of ISAV adjusted for this? From the equation on line 139 it would appear not.

***Author’s response: Lines 126 – 130:*** *Yes, the recovery was adjusted. Retrospective (back) calculations was used to calculate ISAV copy number in 4 or 2.4 ml concentrates by taken into consideration, that ISAV was spiked in 1L of seawater, concentrated and eluted in either 4 or 2.4 ml (lysate) and that from the 4 or 2.4ml, RNA was extracted from 1ml of the lysate. The extracted RNA was eluted in 50 µl and 2 µl of RNA was used for PCR.*

**Reviewer comment:** Line 155: The authors need to explain what they mean by inhibition and how it was quantified and what the threshold was. Additional info also needs to be provided for the ratios 1:1 and 1:4, was this RNA: seawater in the 2ul template (i.e. 1ul RNA + 1 ul seawater = 1:1). Furthermore, and related to the comment above, if RNA was diluted was the yield calculations adjusted accordingly?

***Author’s response: Line 145 – 152:*** *We* *explained inhibition and how it was quantified in the manuscript. Additional, we have also provided detailed information about the dilutions in the manuscript. Environmental samples, such seawater are particularly challenging and usually contain complex mixtures of a variety of inhibitory substances* *that could impact the RT-qPCR and target quantification. For this reason, RNA was therefore, analyzed undiluted (1:1) and diluted (1:4) in duplicates, by RT-qPCR. Potential inhibition was detected when the Cq difference between the 1:1 and 1:4 samples was found to be less than 2 Cq. For the samples that falls within this threshold, the 1:4 dilution was used to estimate virus quantities.* *The ratios 1:1 indicate stock RNA (2 µL template) while the 1:4 ratios was stock RNA: RNA elution buffer (i.e. 1ul RNA + 3 µL RNA elution buffer = 1:4). Yes, when 1:4 dilution was used to estimate virus quantities, virus recovery calculations was adjusted accordingly.*

**Reviewer comment:** Line 165: Three not tree

***Author’s response:*** *Line 165: Corrected from tree to three*

**Reviewer comment:** Line 213: What do the authors mean by ‘reliably detected? Was there a specific threshold used?

***Author’s response:*** *Line 213: The statement ‘reliably detected’ has been rephrased to read “detected”, as there was no threshold used*

**Reviewer comment:** Discussion – I would prefer to have seen some discussion putting the results of this study into the context of what we know about ISAV viral shedding. For example, Gregory et al. 2009 (DOI: 10.1111/j.1365-2761.2009.00999.x) has described the shedding rate and minimal infectious dose of ISAV in experimental challenges and reported their results a TCID 50. In the present study the LOQ was defined, but it remains unclear to this reviewer how this compares to the minimum infectious dose and shedding rate. If the LOQ is higher than the minimum infectious dose then the utility of this assay would be limited. The manuscript would be greatly improved if additional discussion concerning these points could be included.

***Author’s response:*** *We agree with the reviewer suggestion; however, the authors deliberately decided to avoid ISAV virus shedding as we have a separate manuscript on ISAV in experimental challenges study where we focused on ISAV viral shedding by comparing virus recovery from seawater and tissues (gills and heart) over a time period.*
