# Supplementary material for: Short communication: Evaluation of charged membrane filters and buffers for concentration and recovery of infectious salmon anaemia virus in seawater
Source: PLoS One. 2021 Jun 16;16(6):e0253297. doi: 10.1371/journal.pone.0253297 (PMC8208535; doi:10.1371/journal.pone.0253297)
Supplement: Attachment — Submitted filename: Response to Reviewers.docx [file pone.0253297.s005.docx]

**PONE-D-21-01328**

Dear Editor,

Thank you again for the positive feedback, and all the constructive comments to the manuscript: *Evaluation of charged membrane filters and buffers for concentration and recovery of infectious salmon anemia virus in seawater*

**Authors:** Simon Chioma Weli, Haitham Tartor, Bjørn Spilsberg, Ole Bendik Dale, Atle Lillehaug

We have revised the manuscript as recommend by addressing all the comments you raised. We have attached the following files:

1. A revision showing edits made in this revision as tracked changes (in Word doc format), file name: ***“Revised Manuscript with Track Changes***”.
2. A revision with all changes accepted ('clean' file, in Word doc format), which is an unmarked version of the revised manuscript without tracked changes. File labeled ***“manuscript”.***
3. A response letter, file name: ***“Response to Reviewers”***, which is a rebuttal letter that responds to each point raised by the academic editor and reviewers.

**Editor’s comments:** Please double-check the manuscript for similar mistakes (e.g. connected with singular/plural mix-ups). Please double-check the manuscript for similar mistakes (e.g. connected with singular/plural mix-ups).

***Author’s response:*** *As suggested by the editor, we have gone through the manuscript for mistakes.*

**In lines 111 and 114:** Was has been changed to “were used”

**In line 144:** We changed copy number to “copy numbers” and taken has been changed to “by taking”

**In line 166:** We have added such “as”

**In Line 169:** We have removed “therefore”.

**In line 171:** We have changed “falls” to fall”

**In line 173:** We have “ratios were”

**In line 442:** We change Graph show t0 “Graph shows”

We have also reviewed the reference list to ensure that they are complete and correct.
